# Evidence of a health risk ‘signalling effect’ following the introduction of a sugar-sweetened beverage tax

**DOI:** 10.1016/j.foodpol.2021.102104

**Published:** 2021-07

**Authors:** Miriam Alvarado, Tarra L. Penney, Nigel Unwin, Madhuvanti M. Murphy, Jean Adams

**Affiliations:** a Centre for Diet and Activity Research, MRC Epidemiology Unit, University of Cambridge School of Clinical Medicine, Box 285 Institute of Metabolic Science, Cambridge Biomedical Campus, Cambridge CB2 0QQ, United Kingdom; b Global Health Program, Faculty of Health, York University, 4700 Keele Street, Toronto, Canada; c Global Diet and Activity Research, MRC Epidemiology Unit, University of Cambridge School of Clinical Medicine, Box 285 Institute of Metabolic Science, Cambridge Biomedical Campus, Cambridge CB2 0QQ, United Kingdom; d European Centre for Environment and Human Health, University of Exeter Medical School, Knowledge Spa, Royal Cornwall Hospital, Truro, Cornwall TR1 3HD, United Kingdom; e George Alleyne Chronic Disease Research Centre, Caribbean Institute for Health Research, The University of the West Indies, Bridgetown, Barbados

**Keywords:** Sugar-sweetened beverages, Fiscal Policy, Noncommunicable Diseases, Policy evaluation

## Abstract

Consuming sugar-sweetened beverages (SSBs) has been associated with increased rates of obesity and type 2 diabetes, making SSBs an increasingly popular target for taxation. In addition to changing prices, the introduction of an SSB tax may convey information about the health risks of SSBs (a signalling effect). If SSB taxation operates in part by producing a health risk signal, there may be important opportunities to amplify this effect. Our aim was to assess whether there is evidence of a risk signalling effect following the introduction of the Barbados SSB tax. We used process tracing to assess the existence of a signalling effect around sodas and sugar-sweetened juices (juice drinks). We used three data sources: 611 archived transcripts of local television news, 30 interviews with members of the public, and electronic point of sales data (46 months) from a major grocery store chain. We used directed content analysis to assess the qualitative data and an interrupted time series analysis to assess the quantitative data. We found evidence consistent with a risk signalling effect following the introduction of the SSB tax for sodas but not for juice drinks. Consistent with risk signalling theory, the findings suggest that consumers were aware of the tax, believed in a health rationale for the tax, understood that sodas were taxed and perceived that sodas and juice drinks were unhealthy. However consumers appear not to have understood that juice drinks were taxed, potentially reducing tax effectiveness from a health perspective. In addition, the tax may have incentivised companies to increase advertising around juice drinks (undermining any signalling effect) and to introduce low-cost SSB product lines. Policymakers can maximize the impact of risk signals by being clear about the definition of taxed SSBs, emphasizing the health rationale for introducing such a policy, and introducing co-interventions (e.g. marketing restrictions) that reduce opportunities for industry countersignals. These actions may amplify the impact of an SSB tax.

## Highlights

• Sugar-sweetened beverage (SSB) taxes may signal information about health risks.
• Risk signals may be undermined by public misperception of taxed products.
• Industry may produce “countersignals” through advertising.
• Policymakers can amplify positive signalling effects to maximize health benefits.

## 1 Introduction

Sugar-sweetened beverage (SSB) taxation has been recommended as a response to the obesity epidemic (Waqanivalu and Nederveen, 2015). The dominant economic theory around SSB taxation suggests that the introduction of a tax increases prices, which in turn dampens demand and leads to a reduction in sales of taxed products (Mytton et al., 2014). However, it has also been hypothesised that the introduction of an SSB tax may have a ‘signalling effect’ by conveying additional information that prompts behaviour change (Mytton et al., 2014, Smith et al., 2018, Álvarez-Sánchez et al., 2018, Brockwell, 2013, Cornelsen and Smith, 2018, Leicester, 2012, Landon and Graff, 2012). If SSB taxation operates (in part) through a signalling effect, there may be important opportunities to amplify this effect. Cornelsen et al. highlight the importance of understanding “the mechanisms of change in current, implemented, taxes and the role of framing the taxes […] in combination with price changes” (Cornelsen and Smith, 2018). We use the expressive function of law theory to assess signalling as a potential mechanism of change (McAdams, 2015).

### 1.1 Theory: The expressive function of law

McAdams writes in *The Expressive Powers of Law* that laws not only function through sanctions but can also “convey or ‘signal’ information, which affects beliefs and behaviour” (McAdams, 2015). We summarise key elements of this theory below, and then identify implications for SSB taxation.

First, the public must be aware of a law for it to have a potential signalling effect (McAdams, 2015). Second, the way in which the public interprets a law is important. As McAdams elaborates: “Law is not informative […] if they know the law exists, but significantly misunderstand its content” (McAdams, 2015). Third, the ways in which people interpret a law may vary across sub-groups. Finally, the expressive function of law may operate counter to the law’s original intent (McAdams, 2015).

Risk signalling is a particular type of expressive signal, and the strength of a risk signal may be amplified or diminished depending on the public’s perception of lawmakers’ interests. In addition, risk signals may change behaviour both directly (through altered risk perception) and indirectly (through changed social norms). A direct effect may be seen, for example, when a ban on smoking in public places signals new information about risks, leading some smokers to change their behaviour (McAdams, 2015). The same ban could also have an indirect effect, by encouraging non-smokers to be more vocal about their disapproval of smoking behaviour (McAdams, 2015).

This theory has several implications for SSB taxation. First, any risk signal would only apply to the products that consumers perceive as being subject to the tax. Second, the introduction of an SSB tax may be associated with countersignals, such as industry messaging. Third, if lawmakers are perceived to have implemented the SSB tax despite industry opposition, this may strengthen the risk signal (and vice versa). Finally, a risk signal may have direct and indirect effects, by 1) incentivizing consumers to reduce consumption out of concern for their health and 2) encouraging others to voice disapproval of SSB consumption more strongly.

### 1.2 A case study: The Barbados sugar-sweetened beverage tax

We considered the case of the Barbados SSB tax, a 10% tax on sweetened beverages which was initially announced by the Minister of Finance as part of the budgetary presentation (Sinckler, 2015). The Minister of Finance cited high levels of diabetes and the need “to encourage healthier consumption patterns of our people as it relates to the consumption of sweetened beverages” as the rationale for the tax (Sinckler, 2015). According to a nationally representative survey conducted between 2012 and 2013, adult SSB consumption was more than four times the global average (Alvarado et al., 2020) and the prevalence of diabetes was double the global average (Howitt et al., 2015).

There was limited or no public discussion of an SSB tax in the lead-up to announcement (personal communication, August 22, 2017). The tax was announced on June 15th, 2015 and initially planned to take effect on August 1st, 2015, six weeks after being announced (Sinckler, 2015). However, implementation was subsequently delayed to September 2015 (personal communication, January 4, 2017).

We previously evaluated the impact of the tax and found that the 10% tax was associated with a 5.9% increase in the price of SSBs overall (Alvarado et al., 2017) and a 4.3% drop in sales, (Alvarado et al., 2019) based on data from one major grocery store chain.

Our aim in this study was to assess whether there is evidence of a risk signalling effect following the introduction of the Barbados SSB tax.

## 2 Methods

We used process tracing (PT) to evaluate each component of the proposed theory. We present results in terms of updated posterior beliefs, following process tracing best-practice (Beach and Process-Tracing, 2019).

### 2.1 Process tracing as a method

Process tracing is appropriate for single case studies when the research aim is to investigate the presence of a hypothesised causal mechanism in an effort to understand “how and why an intervention led to change” (Beach and Process-Tracing, 2019, Bennett and Checkel, 2014, Punton and Welle, 2015). Process tracing was initially used by psychologists to investigate individual and collective decision-making and later applied in political science (Trampusch and Palier, 2016, George and Lauren, 1979, Ford et al., 1989). More recently, process tracing has been applied in several public health evaluations. For example, Bamanyaki and Holvoet used process tracing to assess gender-responsive budgeting interventions and maternal health service delivery in rural Uganda (Bamanyaki and Holvoet, 2016) and te Lintelo et al. used process tracing to evaluate the impact of the Hunger and Nutrition Commitment Index on international commitments in Bangladesh, Nepal, Malawi and Zambia (te Lintelo et al., 2016).

**Fig. 1:**
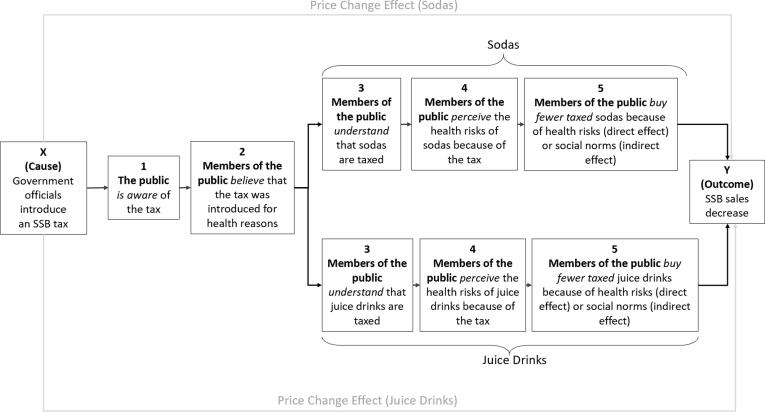
Expressive function of law theory applied to sugar-sweetened beverage (SSB) taxation for sodas and juice drinks separately ^a, b a^ Note that price change represents another mechanism through which the introduction of an SSB tax may influence SSB sales. ^b^ Numbers 1–5 correspond to testable components of the theory; ‘X’ corresponds to the hypothesized cause and ‘Y’ corresponds to the outcome.

The detailed steps and inferential logic of process tracing have been described extensively elsewhere (Beach and Process-Tracing, 2019, Fairfield and Charman, 2017). Briefly, we identified risk signalling (McAdams, 2015) as a plausible mechanism linking the introduction of an SSB tax with reductions in SSB sales (Alvarado et al., 2019). We operationalised this theory to apply to SSB taxation (Fig. 1).

We identified the two most commonly consumed SSBs in the Barbados population: sodas and juice drinks (Alvarado et al., 2020). We assessed the existence of an expressive risk signal around sodas and juice drinks separately, driven by the hypothesis that the risk signal produced by a tax may vary by product type. We refer to sugar-sweetened carbonated drinks (with less than 71 mg caffeine per 12 fl. oz. (Reissig et al., 2009, Kole and Barnhill, 2013)) as ‘sodas,’ drinks containing fruit juice and added sugar as ‘juice drinks,’ and unsweetened juices as ‘no-added sugar juice’ (or ‘NAS juice’). Sodas and juice drinks are taxed under the Barbados SSB tax while NAS juices are not.

For each component of the theorised causal mechanism in Fig. 1, we summarized our prior beliefs based on existing theory, empirical studies and case-specific knowledge (Table 1) (Beach and Process-Tracing, 2019). We then developed a priori predictions by specifying what we would expect to find if each component of the causal mechanism had operated (Table 1).

**Table 1:** Predicted empirical evidence and test types.

Hypotheses (*h*)	Prior p(h)	Part	Means of Verification	Predicted Empirical Evidence (*e*)	Test Type
**(1) The public** is *aware* of the SSB tax	Likely, given evidence of SSB tax awareness in other settings, e.g. 65% awareness in Mexico, (Álvarez-Sánchez et al., 2018) 68% in Berkeley, US (Falbe et al., 2016).	1a	Interviews with members of the public	Participants report being aware of the tax, and can describe details (e.g. when/how they heard about the tax, how it was introduced, etc.)	Hoop Finding *e* does not necessarily confirm *h* given potential bias of participants to report awareness Not finding *e* disconfirms *h*
		1b	Archived media data	Major news sources cover the tax, providing a plausible mechanism for the public to have learned about the policy	Hoop Finding *e* does not necessarily confirm *h* given potential bias of participants to report awareness/news consumption Not finding *e* disconfirms *h* given the news is likely to be the main channel through which people learn about government actions
**(2) Members of the public***believe* that the tax was introduced for health reasons	Agnostic	2a	Interviews with members of the public	Participants report that the tax was introduced because of the health risks of SSBs	Doubly Decisive Finding *e* confirms *h*, Not finding *e* disconfirms *h*
**(3) Members of the public***understand* which products are taxed	Agnostic	3a	Interviews with members of the public	Participants report that the tax is applied on sodas and/or juice drinks	Doubly Decisive Finding *e* confirms *h*, Not finding *e* disconfirms *h*
**(4) Members of the public***increase* their perception of the health risks of SSBs because of the tax	Likely, given evidence of increased newspaper coverage of SSBs as unhealthy in Barbados (Singh-Lalli, 2015).	4a	Interviews with members of the public	Participants mention health risks of sodas and/or juice drinks	Hoop Finding *e* does not necessarily confirm *h* given that participants may be aware of health risks of sodas and/or juices for reasons unrelated to the tax Not finding *e* disconfirms *h*
		4b	Interviews with members of the public	Participants mention increasing their perception of the health risks of sodas and/or juice drinks because of the tax	Smoking Gun Finding *e* confirms *h*, Not finding *e* does not necessarily disconfirm *h* given that participants may not report this so directly (or even be consciously aware of it)
		4c	Archived media data	News media coverage of SSBs as unhealthy increases following introduction of the tax	Hoop Finding *e* does not necessarily confirm *h* given that people may not have seen the news or updated their beliefs based on it Not finding *e* disconfirms *h*
**(5) Members of the public***buy fewer* SSBs based on new information about health risks (direct effect) or social norms (indirect effect)	Agnostic	5a	Electronic point of sale data from a major grocery store chain	Sales of taxed sodas and/or juice drinks decrease over time	Hoop Finding *e* does not necessarily confirm *h* given that other mechanisms could explain the decrease (e.g. price changes due to the tax) Not finding *e* disconfirms *h*
		5b	Electronic point of sale data from a major grocery store chain	Sales of soda/juice drinks *decrease* post-tax, despite *no* tax-driven increases in price, OR Sales of soda/juice drinks *do not* decrease post-tax, despite tax-driven *increases* in price	Hoop Finding *e* does not necessarily confirm *h* given that other mechanisms could explain the decrease/lack of decrease Not finding *e* disconfirms *h*

Note: *e*: evidence; *h*: hypothesis that part of a causal mechanism exists; *p(h)*: probability of the hypothesis being true.

We assessed the probability of finding each piece of evidence if the hypothesis was true, compared to the probability of finding the same evidence if the hypothesis was not true and used this to identify process tracing test types. Briefly, Schmitt and Beach define four test types: straw-in-the-wind tests have low sensitivity and low specificity; doubly-decisive tests have high sensitivity and high specificity; hoop tests have a high false positive rate (low specificity); while smoking gun tests have a high false negative (low sensitivity) (Schmitt and Beach, 2015).

We aimed to specify a test or combination of tests that would allow us to confirm and/or disconfirm each component of the theory, as summarised in Table 1. Where we were only able to identify hoop tests, we developed multiple tests noting that they can, in combination, strengthen confidence in a causal hypothesis (Schmitt and Beach, 2015). When we were able to identify doubly decisive tests, no other tests were necessary given the strong confirmatory and disconfirmatory power of this doubly decisive test.

We analysed relevant data for each test, considering potential biases and limitations in each dataset. We then assessed each component of the hypothesised theory by evaluating the associated empirical tests, guided by test type and prior beliefs. If a test provided evidence in support of (against) a component of the theory we upgraded (downgraded) our prior belief accordingly. Finally, we updated the original theory to reflect our posterior belief in each component of the theory.

### 2.2 Data and analytical methods for empirical tests

We used three pre-existing data sources: archived transcripts of local television news, interviews with members of the public conducted as part of a public acceptability study and electronic point of sale data from a major grocery store chain.

#### 2.2.1 Local news television transcripts

**Fig. A1:**
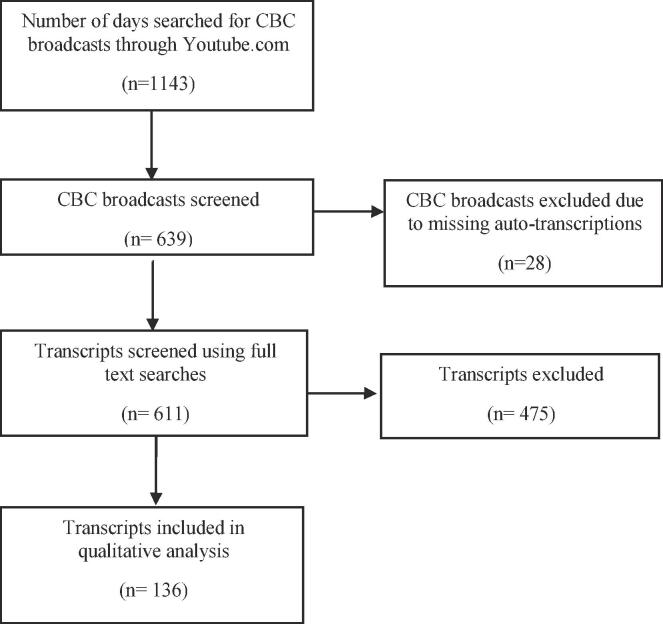
PRISMA-based flow chart around archived media transcripts included in study

We reviewed transcripts of local televised evening news programming from June 15, 2014 (one year prior to announcement of the Barbados SSB tax) to July 31, 2017 (2 + years after tax announcement). The Caribbean Broadcast Corporation (CBC) is the only televised local news programme in Barbados, and some CBC Evening News programmes have been uploaded to the video-sharing website Youtube.com. Televised news footage was available on 639 of the 1,143-day period (56%). Automated text transcriptions were available for all but 28 videos, for a total of 611 news-days (see Appendix Figure A1 for more details). Overall, weekend days were underrepresented and a greater proportion of news-days were available in later years. However, we were not able to identify any systematic pattern in online availability of news programming (other than day of week) which would be likely to systematically bias news content. To address the variable number of news-days available over time, we focused our analysis on changes in the *proportion* of total observed days in each month over time.

Transcripts were analysed using a directed approach to qualitative content analysis (Hsieh and Shannon, 2005). The initial coding scheme was determined by the theory, and included codes such as “SSB tax covered in news” and “SSBs portrayed as unhealthy,” which were reviewed by a senior qualitative researcher (MM). We conducted an initial text keyword search for terms related to SSBs. All results were reviewed and coded, and we explored whether new codes should be developed to capture additional emergent themes. The coded transcripts were then analysed to assess the extent to which they provided confirming or disconfirming evidence of the relevant empirical tests (1b,4c). To assess the frequency of news coverage over time, coded news-days were displayed as a proportion of total observed days in each month over time. All transcripts were coded and analysed in Nvivo 12 Pro, and graphical displays were produced in Stata 14.0 (StataCorp., 2015).

#### 2.2.2 Public acceptability Interviews

In total, 30 participants were recruited for a public acceptability study. Twenty of these participants were recruited from the Health of the Nation (HotN) study, a cross-sectional study conducted between 2012 and 2013 in Barbados. Further details of the original HotN study have been published elsewhere (Howitt et al., 2015). Participants were identified using a stratified sampling procedure based on age, gender and parish of residence. Since the HotN sampling frame did not include participants under the age of 30, ten additional participants between the ages of 18–29 were recruited from a popular local shopping mall. A trained investigator (AF) led semi-structured interviews with each participant around general tax knowledge, views on taxation, and views on the Barbados SSB tax. Written informed consent was obtained from all participants prior to the interview. Interviews lasted 30 min or less, were tape-recorded and transcribed verbatim, and were conducted between March 2017-July 2017. Ethics approval was given by the Research Ethics Committee of the University of the West Indies and Barbados’ Ministry of Health.

Transcripts were analysed using a directed approach and a coding scheme developed from the theory. All transcripts were read initially, then re-read and coded according to the coding scheme. New codes were added to capture emergent themes. The coded transcripts were then analysed to assess the extent to which they provided confirming or disconfirming evidence of the relevant empirical tests. All transcripts were coded and analysed in Nvivo 12 Pro.

#### 2.2.3 Electronic point of sale data

Previously, we assessed trends in sales of carbonated SSBs and non-carbonated SSBs using electronic point of sale data from a major grocery store chain which represented 32% of the grocery store market share in Barbados (personal communication). Data on weekly unit and dollar sales were provided for 1,161 unique size-specific non-alcoholic beverages over 200 weeks, covering the period from January 1, 2013 to October 31, 2016 (including 59 post-tax weeks) (Alvarado et al., 2019). Products were categorized (e.g. as SSBs vs. non-SSBs and as sodas, sugar-sweetened juice drinks, no-added sugar juices, etc.) based on product descriptions which were provided in the original dataset and supplemented by a manual search for product information on manufacturers’ websites and in-store assessments of specific products (Alvarado et al., 2019).

We used an interrupted time series (ITS) analysis and, after controlling for pre-existing trends and time-varying confounders, estimated a 4.3% decline in sales of SSBs following the introduction of the Barbados SSB tax (Alvarado et al., 2019). In this study, we re-analysed trends for soda and juice drinks specifically (our prior categories were broader, e.g. ‘carbonated SSBs’ included highly caffeinated energy drinks and ‘other SSBs’ included sweetened flavoured waters, sports drinks, etc). Additional details on the original analysis are available elsewhere (Alvarado et al., 2019).

Previously, we showed that the year-on-year mean price per litre of SSBs increased in the two quarters following tax implementation, while the price of non-SSBs did not change (Alvarado et al., 2017). In this study, we used a longer time series and revised methodology to assess changes in price in more detail. We conducted ITS analyses separately for soda and juice drinks, using all products with non-missing data over the period from January 2013 to October 2016. We anticipated that prices would change around the time of the tax introduction and then level off, so we pre-specified a step-change only ITS model (i.e. without a post-tax trend effect for price change), following ITS best practice (Bernal et al., 2017). Further details are presented in Appendix Text 1.

We conducted a sensitivity analysis using all products (i.e. including products which were discontinued or introduced during the study period and consequently had ‘missing’ observations for some time points). Given discrepancies between the main model and the sensitivity analysis, we conducted a post-hoc descriptive analysis of post-tax price trends amongst most-sold juice drink brands.

While the tax was initially intended to become effective from August 1, 2015, the actual implementation date was subsequently delayed to September 2015. In both the sales and price change analyses, we used the initial implementation date as the intervention timepoint, considering that companies anticipated paying the tax from this date. We conducted a sensitivity analysis using September 2015 as the intervention date.

## 3 Results

Results of each analysis are presented below, with the corresponding process tracing tests summarised in Table 2. Broadly, we found evidence consistent with the existence of an expressive risk signalling effect around sodas, but not juice drinks.

**Table 2:** Results of empirical tests and implications.

Hypotheses (*h*)	Interpretation (prior → posterior)
**(1) The public** is *aware* of the SSB tax	Likely → Likely It seems likely that people were aware of the tax. However, there is evidence that there were limited reminders of the tax after announcement, which may have dampened any potential risk signal over time.
**(2) Members of the public***believe* that the tax was introduced for health reasons	Agnostic → Very likely It seems very likely that at least some sub-groups believed the tax was introduced for health reasons. However, other sub-groups believed the tax was introduced because of the government’s interest in raising revenue, which may have dampened any potential risk signal amongst this sub-group.
**(3) Members of the public***understand* which products are taxed	Agnostic → Very likely (for sodas) Agnostic → Very unlikely (for juice drinks)
**(4) Members of the public***increase* their perception of the health risks of SSBs because of the tax	Likely → Likely (for sodas) None of this evidence was decisive enough to increase our confidence in whether people increased their perception of sodas as risky following the introduction of the tax. It is also possible that the tax may have re-enforced pre-existing beliefs, and we were not able to rule this out with existing data sources. However, we found no evidence to disconfirm this hypothesis for sodas. Likely → Unlikely (for juice drinks) For juice drinks, the media test was failed, reducing our confidence in this component of the theory. We also found evidence of a potentially unexpected effect: the tax may have incentivised industry to increase advertisements about juice drinks, which may have *reduced* public perception of the health risks of juice drinks.
**(5) Members of the public***buy fewer* SSBs based on new information about health risks (direct effect) or social norms (indirect effect)	Agnostic → Agnostic (for sodas) While the evidence is consistent with this hypothesis for sodas, it is not decisive. For example, an alternative explanation (e.g. that it took time for consumers and markets to adjust to price changes) may be consistent with the observed results Agnostic → Very Unlikely The evidence disconfirms this hypothesis for juice drinks.

Note: e: evidence; h: hypothesis that part of a causal mechanism exist

More specifically, we found evidence consistent with: consumer awareness of the tax (1), consumers’ belief in the health rationale for the tax (2), consumers’ understanding that the tax applied to sodas (3), consumers’ perception that sodas and juice drinks were unhealthy (4), and a reduction in sales of sodas (5). However, our findings reduced our confidence that consumers understood that the tax applied to juice drinks (3) or that the tax was associated with a decrease in sales of juice drinks (5).

Finally, we found evidence that companies may have increased advertising of SSBs and introduced low-cost SSBs in response to the introduction of the tax, potentially undermining a signalling effect.

In the following section, we summarise the evidence for each hypothesis and then present an overall updated version of the theory in Fig. 6.

### 3.1 Were participants aware of the tax? (1a)

The majority of participants reported being aware of the tax. Participants recalled specific sources of information about the tax (e.g. the radio, televised news), and associated the tax with the 2015 budget announcement (Sinckler, 2015). However, some participants were surprised to learn that the tax had already come into effect: “It was implemented yet, though?” (Female, early 30s).

### 3.2 Did popular news sources cover the tax? (1b)

Over a third of participants referred specifically to the CBC local television station provider as a source of information about the SSB tax. When the tax was first announced in June 2015 (Sinckler, 2015), it was covered several times per week on the CBC Evening news. After this initial two-week period, the tax was not mentioned on CBC Evening News from July 2015-October 2016.

Taken together, these two hoop tests suggest that it is likely that consumers were aware of the tax. However, lower awareness of tax *implementation* and lack of subsequent media coverage suggests that any potential signalling effect may have been limited to a specific period.

### 3.3 Do people believe that the tax was introduced for health reasons? (2a)

Participants reported several reasons for the introduction of the tax. Some believed that the tax was primarily about health:

*It was a deterrent to make us stop drinking so many soft drinks. […] (Female, early 40s)*

Other participants suggested that the tax was introduced for both health and revenue reasons:

*The government thought that a way to curb that [childhood obesity], plus make some money on the side is to implement this tax. (Female, mid-20s)*

Some participants suggested that the primary motivation for introducing the tax was to raise revenue and that the health rationale was used to justify the introduction of a new tax:

*I think to get more revenue and then also it has a nice wrapping, a nice story to say ‘oh by the way we help diabetes’ which I mean it will but… (Male, late 20s)*

Some participants reported that the government had acted in its own self-interest by taking advantage of the tax as a revenue-raising opportunity.

This doubly decisive test strongly suggests that at least some (but not all) consumers believed that the tax was introduced for health reasons.

### 3.4 Do participants perceive sodas and/or juice drinks as taxed SSBs? (3a)

Almost every participant who was aware of the tax reported that it was applied to sodas (e.g. “…so like Sprite, Coke, Frutee, Fanta, those kind-a things that are colourful,” Female mid-20s). However, half of participants who knew about the tax reported being unsure about the status of juice drinks:

*I’m not sure if they went on juices as well.* (Male, late teens)

While it was not always clear if participants were referring to NAS juices, colloquial references to “fruit juices” often refer to juice drinks. To further test this, we assessed the context in which juices were discussed in the news media transcripts and found that the phrases ‘juice drinks’ or ‘juices’ were consistently used to refer to taxable, sugar-sweetened juices.

Only a few participants identified juice drinks as taxed SSBs without any prompting. Others reported that juice drinks were untaxed. This doubly decisive test strongly suggests that consumers were aware that sodas were taxed but were mostly unaware that juice drinks were taxed.

### 3.5 Do participants view sodas and/or juice drinks as unhealthy? (4a)

Every participant referred to the health risks of sodas. Specific risks included high levels of sugar (“I guess the bad sugars would be, like, the carbonated drinks and stuff,” Female early-20s) and associations with obesity, diabetes, and chronic diseases more generally:

*Children drinking three and four sweet drinks right now. So by the time them reach adult, twenty-five, some of them done diabetic already. (Female, mid 40s)*

Participants demonstrated a high level of awareness of various health risks associated with soda consumption. In addition, a few participants referred to exerting or experiencing peer pressure around soda consumption because of health-related risks:

*When you’re a big woman like myself and somebody sees you drinking a Coke, they’re going to think, ‘oh, you’re being irresponsible!’ (Female, early 40s)*

This suggests that perceptions of health risks around sodas may have both a direct and indirect effect (social norm enforcement), at least amongst some sub-groups. In comparison, only half of the participants referred to the health risks of juice drinks. Those that did frequently focused on the high sugar content of juice drinks:

*They will show you after they put them to boil and all the water evaporate, how much sugar is left. And the Coke and the Sprite, even the same juices the, they supposed to be health juices ya a big, a big clump of sugar left in it. (Male, early 30s)*

None of these participants linked juice drinks directly with diabetes or other specific health risks beyond containing high sugar levels, and no one referred to social norms around juices. However, some participants suggested that juice drinks were healthy alternatives to sodas:

*It would be more healthier on their bodies ‘cause they more would go to box juice [sugar-sweetened juice drinks sold in small cartons] than more than they would go to a Coke, a Sprite. (Female, mid 40s)*

Overall, there was decisive evidence that participants viewed sodas as unhealthy, passing this hoop test. There was mixed evidence around juices, with some (but not all) participants aware of juice drinks as unhealthy. Even amongst this group however, the link between juices and specific health risks was less clear than it was for sodas.

### 3.6 Do participants view sodas and/or juice drinks as unhealthy because of the tax? (4b)

It was less clear whether awareness of the health risks of sodas was a direct result of the tax. Some participants drew comparisons between SSBs and cigarettes or alcohol, suggesting that SSBs were now in a similar conceptual category as other unhealthy products because of the tax:

*This is perhaps a mechanism being put in place to kind-a curb the consumption of so much sugar. But they want do it with alcohol and the people still drinking. They want do it with cigarettes and people still smoking. But it is a good effort. (Female, mid-30s)*

News coverage of the SSB tax also included references to the similarities between taxation on alcohol and cigarettes:

*The idea is very similar to how we treat alcohol and cigarettes in terms of taxing […] (CBC News, Jan 8, 2017)*

While it is not clear whether people have associated SSBs with alcohol and cigarettes prior to the introduction of the tax, it seems likely that the tax may have either introduced or re-enforced this association, strengthening the health risk signal. One participant suggested that the differential price change between sugar-sweetened and non-sugar-sweetened sodas caused by the tax would signal differences in health risk:

*That [unsweetened club soda] stays at one price but the other ones [regular sodas] go up, then people are obviously going to realise, alright, they sending us a message here. (Male, early 40s)*

One participant suggested that the tax re-enforced existing views of health risks of sodas but did not change existing perceptions of juice as healthy:

*When you think of the tax, you’re going to think soft drink, but you’re not going to think of the Pinehill Dairy juices that you’ve been buying your kids. (Female, early 40s)*

Overall, we did not find strong enough evidence to support the smoking gun test that people changed their views around the health risks of sodas because of the tax (as opposed to re-enforcing pre-existing views). We found no evidence that people changed their views around the health risks of juice drinks.

### 3.7 Did news media coverage of SSBs as unhealthy increase following the introduction of the tax? (4c)

News coverage clearly linking SSBs with specific health risks increased around the time of the tax announcement:

*Literally every home in Barbados has someone that is living with diabetes or they have a friend or they have a co-worker […] people are not educated about what and what sweet drinks can do’ (CBC News, June 26, 2015)*

In the lead-up to the two-year review of the tax, SSBs continued to be portrayed as a health risk:

*A doctor at the forefront of the fight against non-communicable diseases is supporting a proposal for the tax on sweet drinks to be increased. (CBC News, April 19, 2017)*

However, no news coverage focused on health risks of juice drinks specifically, and local terms like “sweet drinks” were dependent on the public’s interpretation. Interviews with members of the public suggest that these vague terms are primarily understood to refer to sodas (3a).

### 3.8 Unexpected expressive consequences of the SSB tax

**Fig. 2:**
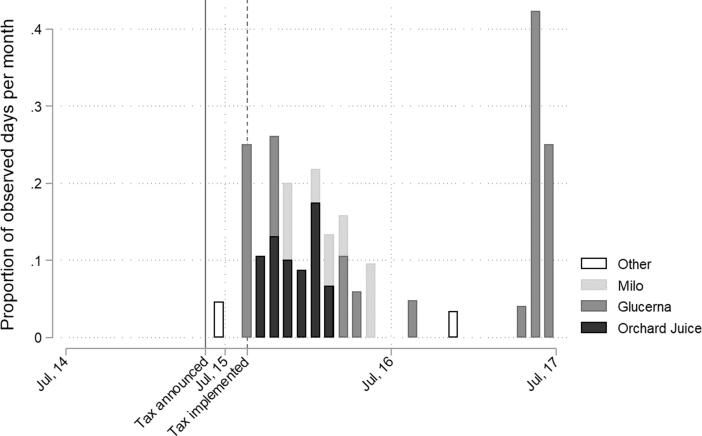
Frequency and distribution of sugar-sweetened beverage (SSB) related advertisements shown during Caribbean Broadcast Corporation (CBC) Evening News, Barbados, June 2014 - July 2017. Note: Orchard is a juice drink brand, Glucerna is a meal-replacement shake targeted at diabetics and Milo is a malt-based drink.

Although not part of the original coding framework, it became apparent from reviewing the archived news transcripts that juice drinks were frequently portrayed on televised advertisements (ads) shown during CBC Evening News programming. We added additional codes and keyword searches to capture SSB-related advertising and summarised the frequency and distribution of these ads over time in Fig. 2.

Given the timing of the increase in these ads, it seems likely that they were introduced in response to the SSB tax. Ad content tended to focus on health benefits of specific SSBs. For example, one ad portrayed juice drinks as a ‘natural choice’:

*Naturally better Orchard. Time for fun, bring out the sun, nutrition so delicious, way less sugar, way less sugar, no artificial sweeteners. Orchard your natural choice. Orchard with real juice…*

The word “natural” was used to describe a sugar-sweetened juice drink four times in this ad. Although emphasising sugar reductions, Orchard juices contained 11.6 g of sugar per 100 mL as of July 2017, making it more sugary than regular Coca Cola which has 10.6 g of sugar per 100 mL.

During interviews with participants, the word “natural” was also used to describe juice drinks as healthy:

*I mean if it’s natural, if it’s, am, orange j, natural orange juice, or so they say, I, I really couldn’t see how the sugar tax would apply to that. […] I know they got drinks with artificial sugars and so on, but…” (Male, early 40s)*

The association between juice drinks and “natural” may have confused some participants’ interpretation of which beverages were taxed (3a):

*Some sugar beverages are supposed to be natural, like fruit juices […] when I hear, when I heard about the sugar-beverage tax, I just study sweet drinks. […] But then when you look at it in-depth you might be, the fruit juices. They will tell you to let you children drink juices rather than, than the sweet drinks, so […] It would be a bit confusing. (Female, mid-40s)*

There is strong emergent evidence that the introduction of the Barbados SSB tax may have inadvertently led to an increase in SSB advertisements, which strongly implied that certain SSBs did not pose health risks (e.g. juice drinks). There is suggestive evidence that these ads may have influenced or re-enforced existing views of juice drinks as healthier than sodas, given the parallels between the advertising messages and participant reflections.

### 3.9 Did consumers buy fewer SSBs? (5a)

**Fig. 3:**
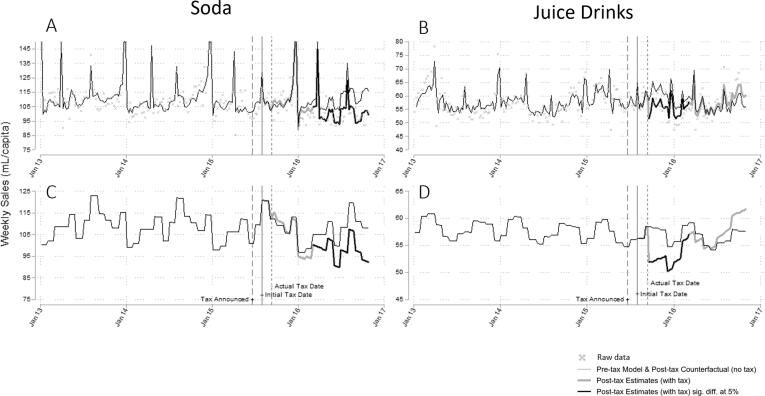
Comparison of sales trends (mL/capita) for sodas and juice drinks, Barbados, 2013–2016 (re-analysis (Alvarado et al., 2017, Alvarado et al., 2019)). Note: The two upper panels (3A and 3B) display the unadjusted model results overlaid with the raw data. Soda sales were truncated at 150 mL/capita for ease of display. The lower panels (3C and 3D) display the model results adjusted for seasonality and holidays.

Sales of sodas decreased in the post-tax period, as shown in Fig. 3.

On average, soda sales changed by −3.8 mL/capita/week [95% CI −4.5 to −3.1] or −3.5% [95% CI −4.2 to −2.9] compared to the estimated counterfactual. By the end of the period, soda sales were 15.8% lower than expected in the absence of the SSB tax [95% CI 5.5 to 26.1].

Sales of juice drinks decreased immediately following the tax and then increased back to pre-tax levels. On average, juice drink sales changed by −1.3 mL/capita/week [95% CI −1.6 to −1.0] or −2.3% [95% CI −2.8 to −1.7] compared to the estimated counterfactual. By the end of the period, juice drink sales were 2.0% higher than expected in the absence of the SSB tax [95% CI −4.2 to 8.3]. Sensitivity analyses using the September 2015 intervention date resulted in slightly larger estimates but did not change the sign or significance of any results.

### 3.10 Do changes in price explain changes in SSB sales? (5b)

**Fig. 4:**
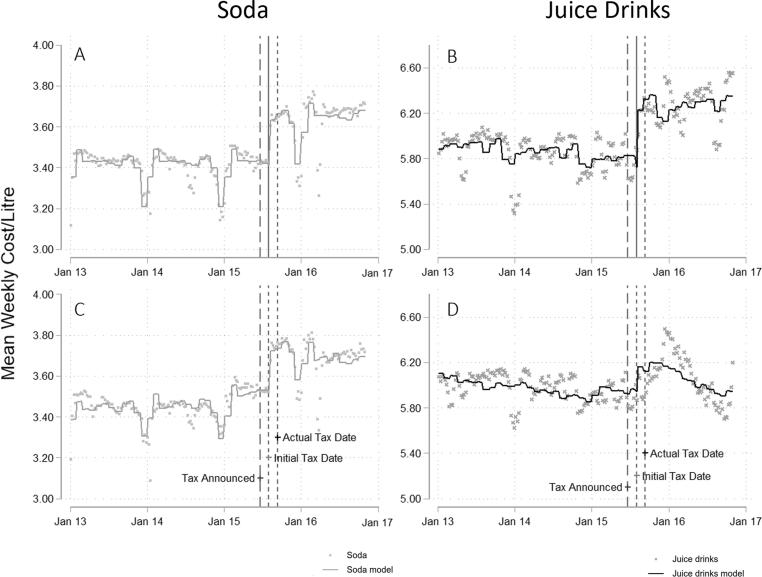
Price of soda and juice drinks following the introduction of the Barbados sugar-sweetened beverage (SSB) tax, 2013–2016, based on products with data in every week of the study period (Panels A and B) and all products (Panels C and D), re-analysis (Alvarado et al., 2017, Alvarado et al., 2019).

The mean consumption-weighted cost per litre increased by 6.2% [95% CI 6.0 to 6.3] for sodas and increased by 9.0% [95% CI 8.8 to 9.2] for juice drinks (see Fig. 4, Panels A and B and Appendix Text 1 and Appendix Table A1). As a sensitivity analysis, we re-estimated the model including all products (regardless of missingness over time). The results were consistent for sodas but varied considerably for juice drinks. For juice drinks, the underlying data suggest that the mean cost per litre increased following the tax and then decreased to below pre-tax levels, as summarised in Fig. 4 Panel D.

This sensitivity analysis suggests that the inclusion of all products substantially changes estimated post-tax trends in juice drink prices. To further explore this, we summarised mean cost per litre for each of the top-selling juice drink brands (which together comprise 75% of sales by volume).

**Fig. 5:**
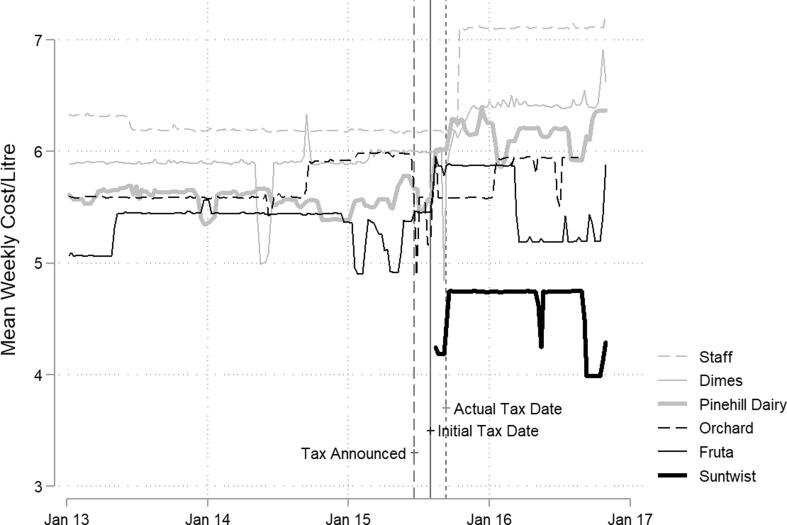
Mean weekly cost per litre, top-selling juice drink brands, Barbados, 2013–2016, (re-analysis (Alvarado et al., 2017, Alvarado et al., 2019)).

We found that a new brand (Suntwist) was introduced around the time the tax was implemented (see Fig. 5). The cost per litre of Suntwist was substantially lower than other top-selling brands (the average post-tax price of Suntwist was 25% lower than the average cost of all other top-selling juice brands). Sales of Suntwist were substantial and increased over the post-tax period, which may explain the post-tax trend for juice drink prices observed in the sensitivity analysis.

Given our interest in consumers’ post-tax purchasing patterns (i.e. including potential purchases of newly introduced products), we focus on results from the sensitivity analysis for the purpose of assessing test 5b.

For sodas, post-tax sales did not seem to respond to price changes immediately (see Fig. 3C and Fig. 4C). Although there was a sharp increase in soda prices around the time of the tax, soda sales remained statistically indistinguishable from the no-tax counterfactual until March 2016. We observed statistically significant reductions in soda sales from March 2016 onwards, despite much smaller absolute fluctuations in price during this time. This provides weak evidence in favour of the hoop test (5b).

In comparison, post-tax trends in sales of juice drinks *do* track price change trends closely (see Fig. 3D and Fig. 4D). Immediately following the tax, prices increased, and sales decreased. Subsequently, mean prices reverted to pre-tax level (and below) and sales increased back to pre-tax levels. This provides evidence against the hoop test (5b), implying that the hypothesis that consumers bought fewer juice drinks because of new health information is unlikely (or suggesting that trends in price change were more important).

### 3.11 Revisiting overall theory

**Fig. 6:**
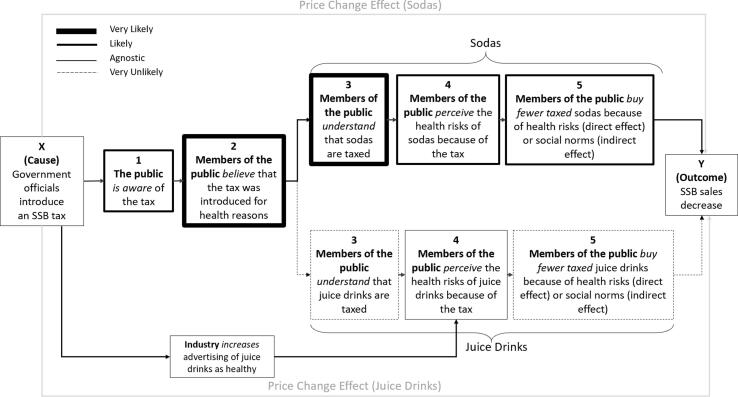
Updated risk signalling theory based on process tracing. Note that our reported levels of confidence do not correspond to the strength of the effect, but rather to the level of confidence we have in each component of the theory after considering the evidence presented above.

Our interpretation of the evidence and updated posterior beliefs are summarised in Table 2 and Fig. 6.

## 4 Discussion

### 4.1 Statement of principal findings

Overall, we found evidence consistent with the existence of a health risk signalling effect following the introduction of the Barbados SSB tax for sodas, but not for juice drinks. We found that consumers were 1) not aware that the tax was applied to juice drinks and 2) unclear about the health risks associated with juice drinks. In addition, we found evidence to suggest that the introduction of the Barbados SSB tax may have incentivised companies to increase advertising around juice drinks as healthy, either re-enforcing existing confusion or counteracting any signalling effect around juice drinks. We also found evidence to suggest that at least one low-cost SSB product line was introduced around the time of the tax introduction, potentially undermining some of the price change effect of the tax.

### 4.2 Strengths and limitations

#### 4.2.1 Related to data sources

This study faced several limitations related to data availability. First, given the short timeframe between the announcement of the tax and implementation (three months), no baseline data on perceptions of different SSBs were collected. Instead, we relied on interview data collected 20–25 months after the implementation of the tax, limiting our ability to assess whether perceptions changed over time.

Second, as with other studies that rely on self-reported data, the interviews that we re-analysed may have been subject to social desirability bias. We identified the tests which may have been most influenced by a social desirability bias (1a, 4a) and interpreted the results of these tests conservatively. However, the empirical tests with the most probative value (2a, 3a, 4b) made use of spontaneously reported descriptions, which are less likely to be biased.

Third, we were not able to access data on the viewership of CBC Evening News programming. However, many participants spontaneously identified CBC Evening News as a source of information about the tax (1a), increasing our confidence in using it as a proxy for news media coverage more broadly. Relatedly, we were only able to access 56% of news-days over our period of interest and it is likely that we missed some relevant content. To address variation over time in news-day availability, we assessed the proportion of news-days per month rather than aboslute number of news-days. It is unlikely that online news-days were systematically missing in a way that related to SSB or tax content.

Fourth, we faced limitations around generalizability within Barbados. For example, although 20 participants for the public acceptability interviews were identified based on age, gender, and parish of residence and the remaining 10 based on age (18–29 years old), our sample was not intended to be statistically representative and may not reflect views across a wider cross-section of the Barbados adult population. Also, the data used for the price and sales analysis were from one supermarket chain, and may not be indicative of price and sales trends in other supermarket or in other retail environments (e.g. gas stations, restaurants, etc.) (Alvarado et al., 2017, Alvarado et al., 2019).

#### 4.2.2 Related to process tracing as a method

There were several strengths and limitations related to our use of process tracing. First, using process tracing encouraged us to identify and use relevant mid-range theory (i.e. the expressive function of law theory (McAdams, 2015)), which allowed us to explore additional levels of nuance around, for example, how consumers understood the law. Second, process tracing provided a transparent and structured framework within which to pre-specify what we would expect to find and critically assess the limitations of each piece of potential evidence. Third, process tracing allowed for inductive insights to be incorporated as new, hypothesised components of theory (e.g. industry responses).

At the same time, using process tracing was very demanding in terms of time and data requirements. In addition, we were able to make stronger claims about eliminating components of the theory (i.e. through failed hoop tests) than we were about confirming components of the theory (i.e. through passed hoop tests). For example, while passing the hoop test about soda price and sales trends (5) provided weak evidence in support of the signalling hypothesis, this evidence did not allow us to eliminate alternative explanations which are also consistent with the observed evidence (e.g. consumers and markets may have needed time to adjust to new prices). This may be a limitation of the kinds of tests we were able to specify and assess, and future process tracing applications may benefit from specifying additional types of tests or narrower hoop tests.

### 4.3 In relation to other studies

#### 4.3.1 SSB taxation

Several other studies have assessed various components of a signalling mechanism following SSB taxation, including awareness of SSB taxation (1) and change in purchases due to new information (5) (Álvarez-Sánchez et al., 2018, Capacci et al., 2017, Brockwell, 2014). At least two evaluations of the Mexico SSB tax explored whether people were aware of the tax (Álvarez-Sánchez et al., 2018, Ortega-Avila et al., 2017). One study found that 65.2% of adults reported being aware of the tax (Álvarez-Sánchez et al., 2018), while a study amongst adolescents found that few participants were aware of the tax (Ortega-Avila et al., 2017), suggesting that awareness may vary considerably by age. We were not able to assess awareness amongst adolescents in this study, but if a similar pattern exists in Barbados this would reduce any potential signalling effect amongst younger age groups.

Other studies have either partially attributed changes in purchases to new information or demonstrated that price change alone does not explain observed trends in purchases (Capacci et al., 2017, Royo-Bordonada et al., 2019). For example, in a sub-national SSB tax evaluation in Catalonia, Spain, participants were asked if they had changed their SSB consumption following the introduction of a tax (Royo-Bordonada et al., 2019). At least some participants (22%) reported “enhanced awareness of their health effects” as the primary reason for having changed their SSB consumption (Royo-Bordonada et al., 2019). However, these results may have been influenced by the study design or social desirability bias. In comparison, we specified intermediary steps and used sales data instead of self-reported data to assess whether purchases changed following tax introduction.

An evaluation of the SSB tax in France (which targeted both SSBs and artificially sweetened beverages) demonstrated that purchases of SSBs decreased even though prices did not change (e.g. the tax was not passed on to consumers) (Capacci et al., 2017). At the same time, purchases of ASBs increased despite tax-driven price increases (Capacci et al., 2017). The authors suggest that this discrepancy is evidence of a signalling effect (Capacci et al., 2017). They relied on a similar test to the one we used (5b), but were able to draw stronger conclusions than we were (i.e. having two failed hoop tests allowed them to draw stronger inferences). Overall, while some studies have assessed various components of the theory we considered here, we are not aware of any studies which have assessed these components collectively in the context of SSB taxation.

#### 4.3.2 Other excise taxes

A number of other studies have assessed the signalling effect of different types of excise taxes (Brockwell, 2014, , 2019, Tiezzi and Verde, 2019, Licari and Meier, 2000). For example, Licari and Meier focused on tobacco taxation and used longitudinal data on cigarette packs consumed per capita by U.S. state from 1955 to 1996. They found that, by the mid-1990 s, the impact of a tax-driven signalling effect was almost as large as the impact of changes in price. They also demonstrated that this signalling effect was lower in tobacco-producing states, whose populations they suggest may be less swayed by government signals about the health risks of tobacco (Licari and Meier, 2000). While they controlled for national policy changes (e.g. advertising bans and warning labels, which may also have signalling effects), they did not control for state-level tobacco control policies which may have coincided with tax changes, potentially confounding the results. Give our single case study design, we were not able to use their approach and instead examined the theoretical mechanisms through which a signalling effect may operate to assess a similar hypothesis.

Finally, Tiezzi and Verde assessed gasoline taxation and found that a gasoline tax has an additional impact on demand, beyond the impact of the tax-inclusive price change (Tiezzi and Verde, 2019). Consumers who were aware that price had changed because of a tax (and not due to other market fluctuations) were observed to reduce demand more than those who are unaware of the tax (Tiezzi and Verde, 2019). The authors suggest this is consistent with the hypothesis that the tax signalled a more permanent price change, implying a higher sustained cost of gasoline consumption. Tiezzi and Verde highlighted that this signalling effect may produce a more regressive outcome, with higher-income households more likely to respond to the additional signal (Tiezzi and Verde, 2019).

This suggests that taxes may produce multiple kinds of signals (permanence of price changes, health risks, etc.) A greater awareness of the health risks associated with SSB consumption may raise the anticipated future costs of current SSB consumption (the internalities of SSB consumption). In the future it would be useful to consider the extent to which anticipated future costs (direct and indirect costs) impact current SSB consumption, following Becker and Murphy’s rationale addiction theory (Becker and Murphy, 1988).

### 4.4 Meaning of the study

We found suggestive evidence that there was a signalling effect around sodas, but no clear signalling effect around juice drinks. This may have been in part because 1) juice drinks were not understood to be taxed, 2) juice drinks were viewed as healthier than sodas (amongst some), and 3) advertising messages (introduced after the tax) emphasised the healthiness of juice drinks. These factors may have re-enforced each other.

### 4.5 Policy implications

There are several broader policy implications that are relevant to all jurisdictions considering implementing SSB taxes, or strengthening existing ones. First, the ways in which consumers interpret a tax (in particular, which products they understand a tax is applied to) may have important implications for the effectiveness of an SSB tax. If this is the case, policymakers, journalists, and health advocates need to be clear about the definition of terms like “sugar-sweetened beverages” and “taxed drinks” and communicate these clearly with the public.

Second, even when a tax is introduced for non-health reasons, it may have a signalling effect if public health groups take advantage of the opportunity to tout health risks (Licari and Meier, 2000). The introduction or amendment of additional SSB taxes for non-health reasons may nevertheless provide strategic opportunities for public health advocates to focus their messaging and amplify the risk signalling potential of any tax change.

Third, introducing or enhancing marketing restrictions may amplify the effect of SSB taxes by reducing ‘strategic’ counter-signalling effects led by industry, particularly around drinks with an existing perception of healthiness. In addition, co-interventions such as front-of-package warning labels may help to reduce confusion around what is a taxed beverage and may re-enforce signalling effects when combined with a tax.

### 4.6 Process tracing

From a methodological perspective, process tracing may be useful when research questions involve testing a theory, rather than assessing the strength of an association, and when the object of study is a large-scale intervention (White and Phillips, 2012). While process tracing guidance centres around developing a linear causal pathway, we suggest that PT could also be used to test components of a more complex non-linear theory.

We suggest that PT may be most valuable, from a public health perspective, when it is used to test causal mechanisms which are less well understood and may be readily intervened upon (e.g. policy-related mechanisms). Finally, while process tracing is not intended to produce generalisable conclusions (Beach and Process-Tracing, 2019), we suggest that the updated theory that process tracing produces may be *analytically* generalizable (Yin, 2013) and can be applied to the evaluation of similar policy interventions in other settings.

### 4.7 Unanswered questions and future research

In the future, it would be useful to assess the extent to which various SSB taxes have operated through a signalling effect and evaluate the impact of variation in signalling strength on changes in SSB sales or consumption. Variation in signalling may be due to how a tax is introduced (Alsukait et al., 2019, Le Bodo et al., 2017, Purtle et al., 2017, Donaldson, 2015, Le Bodo et al., 2019, Thow et al., 2011), media coverage of a tax (Cornelsen and Smith, 2018, Singh-Lalli, 2015, Buckton et al., 2019), co-interventions introduced alongside a tax (Taillie et al., 2020), heterogenous industry reactions (Salgado and Ng, 2019) or contextual factors. Finally, future research around signalling effects could investigate whether there is evidence of variation by sub-groups (e.g. by social class (Smed et al., 2007), age, or habitual consumption level (Li and Dorfman, 2019), which may have an impact on the distributional consequences of SSB taxation.

## 5 Conclusions

We demonstrated that the available evidence is consistent with a health risk signalling effect for sodas following the introduction of the Barbados SSB tax, but not for juice drinks. We found suggestive evidence that some companies may have increased advertising around the healthiness of juice drinks following the introduction of the tax, which may have re-enforced or increased this confusion around juice drinks. We suggest that introducing SSB taxes along with related co-interventions (e.g. front-of-package warning labels, marketing restrictions) may amplify any potential signalling effects by clarifying the health risks associated with specific SSBs.

We build on the expressive function of law theory to describe how a signalling effect may operate following the introduction of an SSB tax and present a refined version of this theory which may be useful for evaluations of SSB taxes in other settings. Finally, we found that applying theory-testing process tracing was a useful approach, with potential applications across an increased range of public health policy evaluations.

## CRediT authorship contribution statement

**Miriam Alvarado:** Conceptualization, Methodology, Software, Formal analysis, Data curation, Writing - original draft, Visualization. **Tarra L. Penney:** Methodology, Writing - review & editing. **Nigel Unwin:** Conceptualization, Methodology, Resources, Writing - review & editing, Supervision. **Madhuvanti M. Murphy:** Methodology, Validation, Resources, Writing - review & editing. **Jean Adams:** Conceptualization, Methodology, Resources, Writing - review & editing, Supervision.

## Declaration of Competing Interest

The authors declare that they have no known competing financial interests or personal relationships that could have appeared to influence the work reported in this paper.

## Figures and Tables

**Table A1:** Post-tax change (%) in average weekly cost per litre (consumption weighted by sales volume).

SSB Type	Products (#)	Coef.	95% CI	
Soda	54	6.2%	6.0%	6.3%
Juice Drinks	48	9.0%	8.8%	9.2%

Note: Regression coefficients and 95% CIs were back-transformed to the original scale using (exp($\beta_{13}$)-1*100) to estimate mean percentage change.

## Appendix

### Appendix Text 1: Price change ITS

First, we conducted a simple ITS analysis, using all products with non-missing data over the period from January 2013 to October 2016, separately for sodas and juice drinks. We anticipated that prices would change around the time of the tax introduction and then level off, so we pre-specified a step-change only ITS model (i.e. without a post-tax trend effect for price change), following ITS best practice (Bernal et al., 2017). When the tax was initially announced (on June 15th, 2015), it was intended to be introduced on August 1, 2015. However, the implementation date was delayed to September 2015. In this analysis, we have used the initial effective date (August 1, 2015), considering that companies would have anticiapted having to pay the tax from this date. However, we display the announcement and actual implementation dates for reference (dashed lines).

To specify the dependent variable, we calculated the consumption-weighted mean weekly log-transformed cost per litre separately for sodas and juice drinks. We used the log-transformed cost per litre since the Barbados SSB tax is an ad valorem tax and we were primarily interested in the percent change in price following the introduction of the tax, rather than the absolute price change. The cost per litre was consumption-weighted to prevent products which are rarely purchased from skewing the analysis. We used the model described in Equation 1:

**Equation 1: Price interrupted time series model**

$${Consumption\;weighted\;mean\;ln(cost\;per\;liter)}_{wy}\sim \;\beta_{1-11}M_{m}+\beta_{12}Trend_{wy}+\beta_{13}Tax_{wy}+\beta_{14}CPI_{my}+\varepsilon_{wy}$$

where *M* corresponds to monthly indicators to take into account seasonality, *Trend* corresponds to the overall linear trend in cost per litre over the whole period, *Tax* corresponds to an indicator for the post-tax period and *CPI* corresponds to the consumer price index (to account for inflation). The coefficient on *Tax* is the coefficient of interest, and 100*(exp($\beta_{13}$)-1) corresponds to the mean estimated percentage change in prices. We summarised the coefficients from this model for sodas and juice drinks in Appendix Table A1, and plot the data and model predictions in Fig. 4A and 3B.

As a sensitivity analysis, we re-estimated both models using all available data per week. However, based on visual inspection it was clear that it the step-change ITS model was not appropriate for juice drinks (see Fig. 4D).

This highlights the impact that excluding products based on missingness over time may have on price change results. To investigate this further, we focused on the top-selling brands for juice drinks (defined by reviewing the top-selling individual products which collectively made up 75% of total sales by volume). We plotted the mean cost per litre by brand over time to descriptively assess whether any top-selling products were introduced or discontinued during this period.

As demonstrated in Fig. 5, at least one major brand was introduced around the same time of the tax introduction. This brand (Suntwist) was 1) popular and 2) low-priced, relative to other juice drinks. The introduction of Suntwist products decreased the average cost per litre faced by consumers, reducing the price impact of the tax on juice drinks. Importantly, Suntwist juice drinks have the same amount of sugar (11.6 g/100 mL) (SunTwist Apple Cherry Fruit Drink, 2020) as other juice drinks or sodas (i.e. a higher sugar concentration than regular Coca Cola at 10.6 g/100 mL).

### Appendix Text 2: Media Transcripts

For a summary of the media transcripts included, see Appendix Fig. A1
